# The genotypic and phenotypic spectrum of MTO1 deficiency

**DOI:** 10.1016/j.ymgme.2017.11.003

**Published:** 2018-01

**Authors:** James J. O'Byrne, Maja Tarailo-Graovac, Aisha Ghani, Michael Champion, Charu Deshpande, Ali Dursun, Riza K. Ozgul, Peter Freisinger, Ian Garber, Tobias B. Haack, Rita Horvath, Ivo Barić, Ralf A. Husain, Leo A.J. Kluijtmans, Urania Kotzaeridou, Andrew A. Morris, Colin J. Ross, Saikat Santra, Jan Smeitink, Mark Tarnopolsky, Saskia B. Wortmann, Johannes A. Mayr, Michaela Brunner-Krainz, Holger Prokisch, Wyeth W. Wasserman, Ron A. Wevers, Udo F. Engelke, Richard J. Rodenburg, Teck Wah Ting, Robert McFarland, Robert W. Taylor, Ramona Salvarinova, Clara D.M. van Karnebeek

**Affiliations:** a Division of Biochemical Diseases, Department of Pediatrics, BC Children's Hospital, BC Children's Hospital Research Institute, University of British Columbia, Vancouver, Canada; b Centre for Molecular Medicine and Therapeutics, Vancouver, BC, Canada; c BC Children's Hospital Research Institute, University of British Columbia, Vancouver, Canada; d Department of Medical Genetics, University of British Columbia, Vancouver, Canada; e Institute of Physiology and Biochemistry, Faculty of Biology, The University of Belgrade, Belgrade, Serbia; f Department of Inherited Metabolic Disease, Guy's and St Thomas' NHS Foundation Trusts, Evelina London Children's Hospital, London, UK; g Clinical Genetics Unit, Guys and St Thomas' NHS Foundation Trust, London, UK; h Hacettepe University, Faculty of Medicine, Institute of Child Health, Department of Pediatric Metabolism, Ankara, Turkey; i Department of Pediatrics, Klinikum Reutlingen, Reutlingen, Germany; j Institute of Human Genetics, Technische Universität München, Munich, Germany; k Institute of Medical Genetics and Applied Genomics, University of Tuebingen, Tuebingen, Germany; l John Walton Muscular Dystrophy Research Centre, Wellcome Trust Centre for Mitochondrial Research, Institute of Genetic Medicine, Newcastle University, Newcastle upon Tyne, UK; m University Hospital Center Zagreb & School of Medicine, University of Zagreb, Croatia; n Centre for Inborn Metabolic Disorders, Department of Neuropediatrics, Jena University Hospital, Jena, Germany; o Department of Laboratory Medicine, Translational Metabolic Laboratory, Radboud University Medical Center, Nijmegen, The Netherlands; p Department of General Pediatrics, Division of Neuropediatrics and Metabolic Medicine, University Hospital Heidelberg, Heidelberg, Germany; q Willink Biochemical Genetics Unit, Manchester Centre for Genomic Medicine, Manchester University Hospitals NHS Foundation Trust, Manchester Academic Health Science Centre, Manchester, UK; r Faculty of Pharmaceutical Sciences, University of British Columbia, Vancouver, BC, Canada; s Department of Clinical Inherited Metabolic Disorders, Birmingham Children's Hospital, Steelhouse Lane, Birmingham, UK; t Radboud Center for Mitochondrial Medicine, Radboud University Medical Center, Nijmegen, The Netherlands; u Department of Pediatrics, Division of Neuromuscular and Neurometabolic Diseases, McMaster University Medical Centre, Hamilton, ON, Canada; v Institute of Human Genetics, Helmholtz Zentrum Munich, Neuherberg, Germany; w Department of Pediatrics, Salzburger Landeskliniken (SALK), Paracelsus Medical University (PMU), Salzburg, Austria; x Department of Pediatrics, Medical University Graz, Graz, Austria; y Genetics Service, Department of Pediatrics, KK Women's and Children's Hospital, Singapore; z Wellcome Centre for Mitochondrial Research, Institute of Neuroscience, Newcastle University, Newcastle upon Tyne, UK; aa Department of Pediatrics, University of British Columbia, Vancouver, BC, Canada; bb Departments of Pediatrics and Clinical Genetics, Academic Medical Center, Amsterdam, The Netherlands

**Keywords:** GDD, global developmental delay, HCM, hypertrophic cardiomyopathy, ID, intellectual disability, MRI, magnetic resonance imaging, MTO1, Mitochondrial tRNA Translation Optimization 1, OXPHOS, oxidative phosphorylation, Q-TOF, quadrupole time-of-flight, WES, whole exome sequencing, Mitochondrial disease, Lactic acidosis, Cardiomyopathy, Ketogenic diet, Mitochondrial translation optimization 1, Oxidative Phosphorylation Defect

## Abstract

**Background:**

Mitochondrial diseases, a group of multi-systemic disorders often characterized by tissue-specific phenotypes, are usually progressive and fatal disorders resulting from defects in oxidative phosphorylation. MTO1 (Mitochondrial tRNA Translation Optimization 1), an evolutionarily conserved protein expressed in high-energy demand tissues has been linked to human early-onset combined oxidative phosphorylation deficiency associated with hypertrophic cardiomyopathy, often referred to as combined oxidative phosphorylation deficiency-10 (COXPD10).

**Material and methods:**

Thirty five cases of MTO1 deficiency were identified and reviewed through international collaboration. The cases of two female siblings, who presented at 1 and 2 years of life with seizures, global developmental delay, hypotonia, elevated lactate and complex I and IV deficiency on muscle biopsy but without cardiomyopathy, are presented in detail.

**Results:**

For the description of phenotypic features, the denominator varies as the literature was insufficient to allow for complete ascertainment of all data for the 35 cases. An extensive review of all known MTO1 deficiency cases revealed the most common features at presentation to be lactic acidosis (LA) (21/34; 62% cases) and hypertrophic cardiomyopathy (15/34; 44% cases). Eventually lactic acidosis and hypertrophic cardiomyopathy are described in 35/35 (100%) and 27/34 (79%) of patients with MTO1 deficiency, respectively; with global developmental delay/intellectual disability present in 28/29 (97%), feeding difficulties in 17/35 (49%), failure to thrive in 12/35 (34%), seizures in 12/35 (34%), optic atrophy in 11/21 (52%) and ataxia in 7/34 (21%). There are 19 different pathogenic *MTO1* variants identified in these 35 cases: one splice-site, 3 frameshift and 15 missense variants. None have bi-allelic variants that completely inactivate MTO1; however, patients where one variant is truncating (*i.e.* frameshift) while the second one is a missense appear to have a more severe, even fatal, phenotype. These data suggest that complete loss of MTO1 is not viable. A ketogenic diet may have exerted a favourable effect on seizures in 2/5 patients.

**Conclusion:**

MTO1 deficiency is lethal in some but not all cases, and a genotype-phenotype relation is suggested. Aside from lactic acidosis and cardiomyopathy, developmental delay and other phenotypic features affecting multiple organ systems are often present in these patients, suggesting a broader spectrum than hitherto reported. The diagnosis should be suspected on clinical features and the presence of markers of mitochondrial dysfunction in body fluids, especially low residual complex I, III and IV activity in muscle. Molecular confirmation is required and targeted genomic testing may be the most efficient approach. Although subjective clinical improvement was observed in a small number of patients on therapies such as ketogenic diet and dichloroacetate, no evidence-based effective therapy exists.

## Highlights

• The phenotypic and genotypic spectrum of MTO1 deficiency is more variable than previously reported
• Hallmark features: cardiomyopathy, lactic acidosis, developmental delay, failure to thrive, seizures, optic atrophy, ataxia
• 19 pathogenic *MTO1* mutations (splice-site, frameshift, missense) were identified with a geno-phenotype relation
• Suspect MTO1 deficiency based on clinical features, mitochondrial markers in body fluids, low complex I III IV activity
• Although ketogenic diet exerted subjective improvement in some cases, there is exists no evidence-based effective therapy

## 1 Introduction

Mitochondrial diseases encompass a broad range of disorders that may result from pathogenic mutations of mitochondrial DNA or the nuclear genome and affect all ages in a mono- or multisystemic manner with an estimated prevalence of ~ 1:5,000 [1], [2]. Approximately 1200 different nuclear genes encode mitochondrial proteins and causal defects have been identified in ~ 300 genes, many through genome wide sequencing approaches [3], [4]. Continued identification of nuclear genes associated with mitochondrial disease will result in a greater understanding of mitochondrial homeostasis and function [5].

A novel form of mitochondrial disease caused by mutations in *MTO1*, encoding the mitochondrial tRNA translation optimization 1 (MTO1) protein (OMIM#614667) was first described in 2012 in 2 Italian siblings, born of unrelated parents, presenting soon after birth with lactic acidosis, severe hypoglycaemia and fatal infantile hypertrophic cardiomyopathy [6], [7]. MTO1 is one of two subunits of an enzyme that catalyzes the 5-carboxymethylaminomethylation of the wobble uridine base in the mitochondrial tRNAs specific to leucine, tryptophan, glutamine, glutamic acid and lysine [8], [9], [10]. This post-transcriptional modification is critical to the accuracy and efficiency of mtDNA translation. As is observed with other nuclear-mitochondrial diseases of mt-tRNA modification such as TRIT1, TRMT5, GTPBP3 and PUS1, MTO1 deficiency usually results in a combined reduction of mtDNA-dependent respiratory chain activities [6], [11], [12], [13], [14].

Hallmark features of MTO1 deficiency include hypertrophic cardiomyopathy (HCM), lactic acidosis (LA) and mild to severe global developmental delay (GDD)/intellectual disability (ID) [6], [15] but significant phenotypic heterogeneity exists. Two female siblings with MTO1 mitochondriopathy who remain without CM at the adolescent age are presented in detail in this paper. A mouse model of the MTO1 deficiency was recently generated, and shown to have similar to human phenotype [16]. Using the mouse embryonic fibroblasts from the MTO1-deficient mice, it was recently demonstrated that MTO1 controls mitochondrial translation rate via mt-tRNA modification in a tissue-specific manner [11]. This explains the organ specific pathologies and the reported phenotypic heterogeneity. The aim of this study was to review and report on 35 cases of MTO1-deficient mitochondrial disease, outlining their clinical and molecular features, response to treatment and genotype-phenotype evaluation.

## 2 Cases and methods

### 2.1 Clinical descriptions

Two female siblings and their parents were enrolled into the Treatable Intellectual Disability Endeavour eXome (TIDEX) neurometabolic discovery study (UBC IRB approval H12-00067) [17]. Written consent for data and sample collection, whole exome sequencing (WES), as well as publication of the current case reports was provided by the parents. Consent for publication of the other cases mentioned in the tables was provided to the clinician or laboratory scientist at the respective institutions. These naturally conceived children were born to non-consanguineous parents of Caucasian (paternal) and Malayo-Polynesian ethnicity (maternal). Family history was significant for a maternal uncle with ID, a maternal aunt and first cousin with a history of febrile seizures, and a maternal first cousin with speech delay and hyperactivity.

*Case* 1: this 16-year-old female presented with early onset mild developmental delay and atypical febrile seizure at 2.5 years with subsequent absence seizures at 3.5 years. She was born at 38 weeks gestation, by spontaneous vaginal delivery, weighing 3.34 kg. She sat unsupported at 8 months of age and started to cruise at 12 months. First concerns were raised with speech delay (first words were at 2 years). Physical exam at age 4 years showed hypotonia with moderate delay in gross motor, fine motor, expressive language (approximately 100 words) and social skills. Plasma and CSF lactate levels were elevated at 4.4 mmol/L (ref range: 0.5–2.2 mmol/L) and 3.3 mmol/L respectively. Plasma amino acids demonstrated increased alanine of 657 μmol/L (reference range 148–475 μmol/L). Respiratory chain analysis on muscle biopsy showed complex I and IV deficiency (complex I activity: 4.1 nmol/min/mg (ref range 17.9–56.7); complex IV activity: 0.76 nmol/min/mg (ref range 2.3–5.03)). Pathological analysis of the muscle demonstrated a greater number of type 1 than type 2 fibres with variation in fibre size diameter but no histological, histochemical or ultrastructural features to suggest a mitochondrial myopathy. She was commenced on oral L-carnitine, thiamine and riboflavin, with coenzyme q10, vitamin E, and vitamin D added later.

At age 6 years, the patient was ataxic with generalized muscle weakness, was unable to ride a tricycle, and could only make 2–3 word sentences. She experienced behavioural change with the onset of aggressiveness and pica. At 7 years a ketogenic diet was started (highest ratio 4.75:1) which initially reduced her seizure activity, but six months after commencement, in the context of a severe flu-like illness, she developed a sudden reduction in bilateral visual acuity (10/400), ptosis and generalised weakness. Fundoscopy demonstrated bilateral optic disk pallor, localized to the papillomacular bundle. MRI brain was normal at 3 and 8 years, but at age 9 years a signal abnormality in the midline of the brain and in the optic chiasm was detected on T2 imaging. At 11 years she presented in status epilepticus and CT/MRI brain imaging demonstrated multiple areas of hyperintense T2/FLAIR signal within the cerebral peduncles, basal ganglia and cortex most likely due to a metabolic crisis which had largely resolved one week later. Seizures proved refractory to treatment and the ketogenic diet was reduced to a 2:1 ratio at age 12 years. This was followed by an improvement of her visual acuity and subjective energy levels.

The phenotype evolved and assessment at 16 years of age showed moderate ID requiring a special life skills program in school, optic neuropathy with good visual acuity in both eyes, bilateral ptosis, mild facial weakness, myopathic ataxia with mild dysmetria with intention tremor and dysarthria with decreased muscle mass and proximal muscle strength but without bulbar symptoms and constipation. Echocardiography (ECHO) was unremarkable at age 15 years. Short stature between ages 8 to 15 years (height less than 3rd centile and weight on the 3rd centile), prompted an endocrinology work-up which showed no evidence of hormonal abnormalities and at 16 years her height and weight were normal (3rd–10th centile). Head circumference remained normal (between mean and + 1 SD). The patient’s seizures were poorly controlled and she continued to have up to 40 seizures per week on a ketogenic diet (2:1), levetiracetam, piracetam, clonazepam and rufinamide which varied from generalized, absence, to drop seizures. Hearing assessment was normal.

*Case* 2 is a 12-year-old full female sibling who presented with a similar albeit milder phenotype, with febrile seizures at 19 and 26 months and subsequently developed afebrile seizures. She was born at 40 weeks gestation, by spontaneous vaginal delivery, weighing 3.85 kg. During the pregnancy, maternal gestational diabetes was present which required insulin from 7 months gestation. At 3.5 years elevated plasma lactate (3.2–4.2 mmol/L; ref range 0.5–2.2 mmol/L) and alanine (711 μmol/L; ref range 148–475 μmol/L) were noted. At 3 years the patient was considered developmentally appropriate but at 4.5 years she required aid in preschool. She was dysarthric with generalized weakness, and tended to trip when walking. At 4 years she started on oral riboflavin, coenzyme Q10, L-carnitine. Cranial MRI/spectroscopy and electromyography/nerve conduction studies were normal. Respiratory chain analysis on muscle biopsy showed complex I and IV deficiencies (complex I activity – 11.3 nmol/min/mg (ref range 17.9–56.7) and complex IV- 02.16 nmol/min/mg (ref range 2.3–5.03)). Pathology, including histochemistry and electron microscopy was normal.

Ophthalmological and audiological exams as well as ECHO were normal at 5 and 12 years respectively. At 12 years, she showed moderate ID and attended mainstream school with an adapted program. She had normal muscle strength in her face and limbs with mild hypotonia and no bulbar symptoms. Her deep tendon reflexes were normal. Her seizures varied and were well controlled with clobazam, levetiracetam, lamotrigine and ethosuximide. Hence the modified Atkins diet was not attempted. Growth parameters were consistently normal with height and weight between the 90th–95th centile and her head circumference between 25th–50th centile. For a clinical summary of these two cases see patient numbers 4 and 5 of Table 1.

**Table 1:** Overview of the clinical data of patients with confirmed MTO1 deficiency.

Pat. No.	M/F	Ethnicity +/- Consang (C or NC)	Age of Presentation (days/yrs)	Presenting feature (s)	Current age/age at publication (yrs)	Clinical features												
						Psychomotor delay				CNS/muscular			Cardiac disease		Optic pathology	FTT, feeding difficulties, others	Brain MRI/MRS	
						ID/GDD	GMD	FMD	SD	SZ	Hypotonia/dystonia/ataxia	Weakness	CM	Abnormal Rhythm			MRI	MRS
1	F	Caucasian NC	2 yrs	DD, seizures; visual deterioration; ptosis, ataxia, hypotonia, myopathy	16	Y	Y	N	Y	Y	Hypotonia	Y	N	N	Visual deterioration	FTT, FD	Ab	NR
2	F	Caucasian NC	2 yrs	DD, seizures, hypotonia, myopathy	12	Y	Y	N	Y	Y	Hypotonia Ataxia	Y	N	Y	N	FTT, FD	NL	NL
3	M	Syrian C	Day 1	LA, hypocalcaemia, resp. distress	4.41	Y	Y	Y	Y	N	Hypotonia Ataxia	Y	HCM	N	N	FTT, FD	Ab	Ab
4	M	Caucasian NC	Day 2	Lactic acidosis mild tubulopathy Resp. distress	1.08	Y	Y	Y	N	N	Hypotonia	Y	N	N	ND	FTT, FD, Tubulopathy	Ab	NR
5	F	Caucasian NC	Day 1	LA, SGA, dysmorphic features	8.9	Y	Y	Y	Y	Y	Hypotonia	Y	HCM	WPW	Ophthalmopl-egia	FTT, FD Terminal deletion chr 8 (P arm)	Ab	NR
6	F	Turkish NC	Day 2	Drowsiness, ataxia, fatigue, seizures, cardiac murmur, hepatomegaly	12.33	Y	Y	Y	N	Y	Hypotonia Ataxia	Y	HCM	Y	N	FTT, FD Autistic spectrum disorder Hepatomegaly	Ab	NR
7	F	Pakistan C	Day 2	HCM, LA	Deceased at 0.66yr	Y	Y	Y	NA	N	Hypotonia	Y	HCM	N	Mottling of retinal pigment epithelium	FD	NL	NL
8	M	Caucasian NC	0.58 yrs	HCM, LA, DD, Hypotonia,	5.83 yr	Y	Y	Y	Y	N	Hypotonia Ataxia	N	HCM	N	N	FTT, FD	Ab	NR
9	F	Caucasian NC	0.167 yrs	HCM, LA, hypotonia	Deceased at 0.66 yr	Y	Y	Y	NA	NR	Hypotonia	NR	HCM	N	NR	FTT, FD	Ab	NR
10	F	Croatian NC	Day 1	HCM, muscle, CNS	Deceased on day 2	NR	NR	NR	NR	N	Hypotonia	Y	HCM	N	NR	NR	NR	NR
11	M	British -Pakistani C	0.08 yr	HCM, muscle, CNS; LA	deceased at 0.08 yr	-	-	-	-	N	Hypotonia	N	HCM	N	N	N	NR	NR
12	M	British-Pakistani C	1 yr	DD, microcephaly, hepatosplenomegaly	deceased at 2.75 yr	Y	Y	Y	Y	N	Hypotonia	Y	DCM	NR	NR	Hepatic involvement	NL	NR
13	M	British NC	0.5 yr	Muscle, lactic acidosis	2	Y	NR	NR	NR	NR	Hypotonia	Y	N	NR	NR	NR	NR	NR
14	M	European (Italy) NC	Day 1	LA, HCM, hypoglycemia, hyperammonemia (mild)	Deceased 0.052 yr	NR	NR	NR	NR	NR	NR	NR	HCM	Y	NR		NR	NR
15	F	European (Italy) NC	Day 1	LA, HCM, hypotonia	Deceased at 0.110 years	NR	NR	NR	NR	NR	NR	NR	HCM	Y			NR	NR
16	M	European (Italy) NC	0.08 yr	HCM; LA, FD, weakness, reduced ocular fixation	20 + yr	N	N	Y	N	N	N	N	HCM	Y	bilateral optic atrophy and increased P100 latency	FD Hepatic involvement	NL	NR
17	F	European (Italy) NC	Day 2	LA; hypotonia, dystonia	14 yr	Y	Y	NR	Y	Y	Hypotonia/dystonia	NR	HCM	N	abnormalities of visual and brainstem evoked potentials and nerve conduction velocities.	FTT, FD	Ab	NR
18	M	Pakistan C	Day 1	FTT, FD, hypotonia	Deceased at 1 yr	Y	Y	Y	NR	N	Hypotonia	NR	HCM	N	NR	FTT; FD	Ab	Ab
19	M	Pakistan C	Day 1	HCM, FTT, FD, hypotonia.	Deceased at 0.25 yr	NR	NR	NR	NR	N	Hypotonia	NR	HCM	NR	NR	FTT, FD	NR	NR
20	F	Pakistan C	0.25 yr	LA, HCM, encephalopathy, seizures; fatigue	19 yr	Y	Y	NR	Y	Y	NR	NR	HCM	NR	NR	Encephalopathy Vitamin D deficiency	NR	NR
21	F	Pakistan C	0.42 yr	LA, HCM, WPW; DD	12 yr	Y	Y	Y	Y	N	NR	Nr	HCM	WPW		Pericardial effusion	NR	NR
22	M	NR C	NR	HCM, DD, hypotonia, dysdiachokinesia,	15 yr	NR	NR	NR	NR	NR	NR	NR	HCM	NR	NR	NR	NR	NR
23	F	Caucasian NC	0.038 yr	LA, tachypnoea	deceased at 23 yr	Y	Y	Y	Y	Y	Hypotonia	Y	N	N	Optic atrophy	FD	Ab	NR
24	F	Caucasian NC	Day 1	LA, hypoglycaemia (persistent)	22 yr	Y	Y	Y	Y	N	Hypotonia	Y	HCM	N	Optic atrophy	FD	Ab	NR
25	M	Turkish NC	0.42 yr	LA (infection)	13.83 yr	Y	Y	Y	Y	N	Hypotonia/ataxia	Y	HCM	N	N	FTT	Ab	Ab
26	M	Turkish NC	0.08 yr	LA (infection)	deceased at 3.25 yr	Y	Y	Y	Y	N	Hypotonia/ataxia	Y	HCM	N	N	FTT, FD	NR	NR
27	M	Caucasian NC	Day 1	HCM, tachypnea, multiorgan failure (cardiac, pulmonary, renal)	deceased at 0.005 yr	NA	NA	NA	NA	Y	Hypotonia	NR	Unknown	unknown	NR	Hyperpnea; pulmonary edema Renal failure	NR	NR
28	M	Caucasian NC	Day 1	Tachypnea, somnolence	deceased at 0.043 yr	NA	NA	NA	NA	N	NR	NR	HCM	NR	NR	Hyperpnea, FTT	NR	NR
29	M	European (Spain) NC	0.5 yr	FTT, DD, hypotonia, truncal ataxia	16 yr	Y	NR	NR	NR	Y	Ataxia	Nr	HCM	NR	Optic atrophy		NL	NR
30	F	European NC	8 yr	ID, clumsiness, fatigability	19 yr	Y	Y	NR	NR	N	N	NR	HCM	tachycardia	bilateral partial papillary atrophy		Ab	NR
31	F	European (Germany) NC	Day 1	LA	NR	Y	Y	NR	Y	Y	NR	NR	HCM	N	Optic atrophy		Ab	NR
32	F	NR C	0.5 yr	FTT	30 yr	Y	NR	NR	NR	Y	NR	NR	HCM	NR	Optic atrophy, severe myopia	FTT Dorsal kyphosis	NR	NR
33	M	NR C	5 yr (but DD noted from young age)	Fatigue, HCM	28 yr	Y	NR	NR	NR	NR	NR	NR	HCM	NR	Optic atrophy	Dorsal kyphosis	NR	NR
34	F	India C	Day 1	LA, tachypnoea	0.42 yr	Y	Y	Y	NA	N	Hypotonia	N	HCM	N	N	FD	NL	NL
35	F	European (Germany)	6 yr	Seizures	22 yr	Y	N	N	N	Y	N	N	N	N	Optic Atrophy, bilateral cataracts	N	Ab	NL

Detailed phenotype of 35 patients with MTO1 deficiency including, sex, ethnicity, age at presentation, presenting features and eventual clinical features including neurological, cardiac, ophthalmic pathology and Brain MRI/CT). Ab: Abnormal; C: Consanguineous; DD: developmental delay; FD: feeding difficulties; FTT: failure to thrive; HCM: hypertrophic cardiomyopathy; LA: lactic acidosis; N: no; NL: normal; NC: non-consanguineous; NR: not recorded; SGA: small for gestational age; SZ: seizures; WPW: Wolf Parkinson White; Y: yes.

### 2.2 Whole exome sequencing for the two presented siblings

Quad-WES analysis (index-affected sibling-unaffected mother-unaffected father) using the Agilent SureSelect kit and Illumina HiSeq 2000 (Perkin-Elmer, USA) was performed. The data was analysed using our semi-automated bioinformatics pipeline [17]: (1) the sequencing reads were aligned to the human reference genome version hg19 using Bowtie 2 [18], (2) the duplicates were marked and sorted using Picard, (3) variants were called using SAMtools and BCFtools after indel realignment using GATK, (4) transcripts were annotated using snpEff [19], (5) functional variants were prioritized for rare variants by comparison against the public databases [dbSNP, NHLBI Exome Sequencing Project Exome Variant Server, and Exome Aggregation Consortium (ExAC)] and (6) subsequently screened under a series of Mendelian inheritance models: homozygous, hemizygous, compound heterozygous and *de novo* as described previously [20].

### 2.3 Identification and review of 35 cases of MTO1 deficiency

Through a PubMed literature search (2012–2017) and direct contact with clinical colleagues caring for mitochondrial diseases patients, we identified and collected clinical and genotypic information on a further 33 patients (18 published and 15 unpublished) with pathogenic mutations in *MTO1*. The data on the total of 35 patients were collated in Excel. Statistical analysis was limited to descriptive and confidence interval calculations (https://www.pedro.org.au/english/downloads/confidence-interval-calculator/).

## 3 Results

### 3.1 WES analysis for the two female siblings and parents

**Supplementary Fig. 1:**
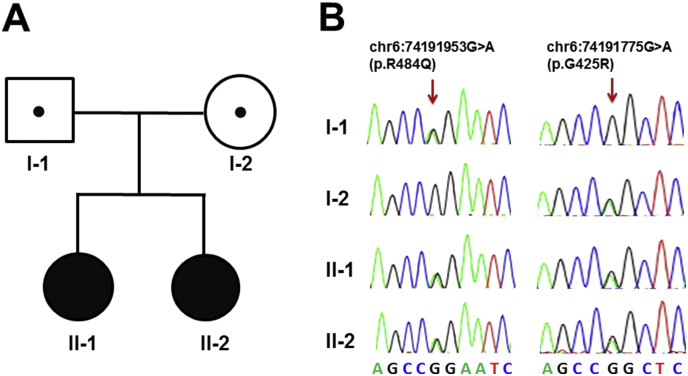
Pedigree of Cases 1 and 2 and sanger sequencing results of the MTO1 alleles confirms the exome sequencing data.

Whole exome sequencing (WES) identified three candidate genes (*CDH23*, *C5orf42* and *MTO1*), all three with compound heterozygous variants. Of these three candidate genes, the variants on chromosome 6 affecting the MTO1 protein were deemed to be the best explanation for the observed phenotype in the siblings. Both affected siblings are compound heterozygous (Supplementary Fig. 1) for two variants (c.1451G > A; p.Arg484Gln) and (c.1273G > A; p.Gly425Arg) in *MTO1* (NM 012123.3; NP 036255) on chromosome 6 which are predicted *in silico* to be damaging by all tested tools. One of the variants, p.Arg484Gln, is novel and had not been observed in analysed databases, including dbSNP (v.142), NHLBI ESP, in – house or ExAC while the second variant, p.Gly425Arg, had been reported once in the ExAC database (Table 2). Sanger sequencing confirmed segregation with disease according to the recessive inheritance model, with carrier status in each parent.

### 3.2 Clinical phenotypic spectrum (Table 1 and Supplementary Table 1)

Despite best efforts complete data for all cases was not possible to ascertain which has resulted in a variation of the denominator from 35.

An overview of clinical features of the 35 patients (17 males and 18 females) from 11 different countries (Austria, Canada, Croatia, Germany, India, Italy, Pakistan, Turkey, Spain, Syria and the United Kingdom) is presented in Table 1.

12/35 (34%) cases are offspring to consanguineous parents. 15/34 (44%) presented in the first 2 days of life. The average age of presentation was 10.2 months (range: day 1 to 8.0 years).

At the time of writing 12/35 (34%) patients were deceased with an average age of death of 2.67 years (range: week 1 to 23 years). 8/17 (47%) of males passed away at an average age at death of 0.96 years (week 1 to 3.25 years). 4/18 (22%) of females were deceased with an average age at death of 6.1 years (7 months to 23 years). Of those presenting clinically in the first 2 days of life, 7 patients (47%) passed away before the age of 2 years, suggesting a relationship between earlier presentation and a poorer prognosis.

The most common clinical feature at initial presentation was hypertrophic cardiomyopathy (HCM), present in 15/34 [44%; 95% CI, 29–60%] cases which over time developed in 27/34 [79%; 95% CI, 63–90%] cases. Of note one patient (patient no.12) developed dilated CM which may have been HCM at an earlier stage. Hypotonia was identified in 10/35 [29%; 95% CI, 16–45%] cases at presentation but it eventually occurred in 22/35 [63%; 95% CI, 46–77%] cases. GDD/ID affected 28/29 [97%; 95% CI, 83–99%] cases for whom this information was available; one patient was described to have normal scholastic performance at age 19 years. Failure to thrive (FTT) was reported in 12/35 [34%; 95% CI, 21–51%] cases, and feeding difficulties in 17/35 [49%; 95% CI, 33–64%] cases. Optic atrophy occurred in 11/21 [52%; 95% CI, 32–72%] cases with thinning of the retinal nerve fibre layer documented in three of these cases. Other ocular pathology noted in this case series included external ophthalmoplegia (1/20 cases), unilateral ptosis (1/20 cases) and mild bilateral cataracts (1/20 cases). Seizures were not a frequent initial symptom (only present in 5/35 [14%; 95% CI, 6–29%] but developed over time in 12/35 [34%; 95% CI, 21–51%] cases. Ataxia was diagnosed in 3/34 [9%; 95% CI 3–23%] cases at initial presentation and during follow-up in 7/34 [21%; 95% CI, 10–37%]. Hepatic dysfunction was infrequent, present in 3/35 [9%; 95% CI, 3–22%]. Renal involvement was also infrequent (1/35 [3%; 95% CI 0.5–14%]). Renal dysfunction, present in patient 12 is thought to be secondary to CM and poor perfusion.

All cases of MTO1 deficiency presented with a combination of the above described clinical features. There was no documented history of diabetes or ptosis and only one documented case of hypoglycaemia.

Cranial MRIs were reported normal in 6/20 patients in whom it was performed [30%; 95% CI, 15–52%]. Documented abnormalities in the 14/20 [70%; 95% CI, 48–85%] cases included lesions of the basal ganglia and cerebellar peduncles and hypoplasia of the corpus callosum. An elevated lactate peak on brain scan was identified in 2/5 cases in whom spectroscopy was performed. (Table 1 and Supplementary Table 1)

### 3.3 Genotypic spectrum (Table 2)

**Fig. 1:**
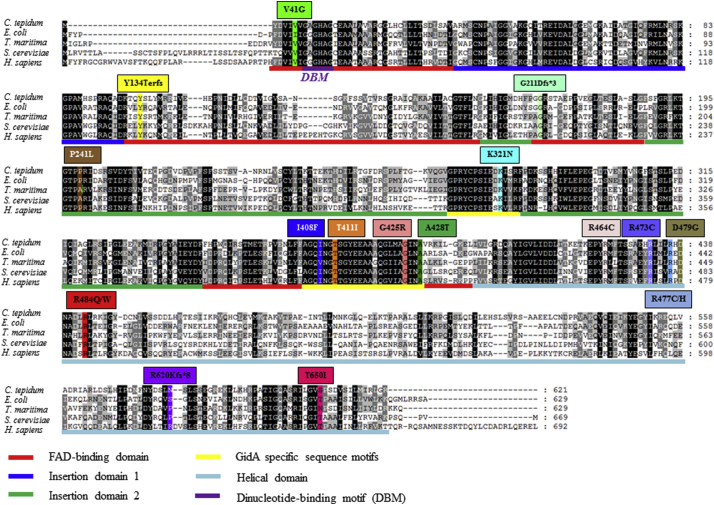
Protein sequence alignments of human MTO1 protein with orthologs. Sequence alignment of the human MTO1 protein with orthologs from *Chlorobium tepidum*, *Escherichia coli*, *Thermotoga maritima* and *Saccharomyces cerevisiae*. The conserved amino acids are shaded and identified mutations in the cohort of 35 patients are denoted. The domain and motif locations are depicted by coloured lines according to previously published work by Meyer et al. [21].

In the 35 patients from 26 unrelated families, we identified 19 different *MTO1* (NM 012123.3; NP 036255) variants (7 published and 12 unpublished): 15 missense, 3 frameshifts and one splice-site (Table 2 and Fig. 1). Please note that for consistency and proper genotype-phenotype comparisons, we re-annotated all variants, including those previously published, according to the NM 012123.3 transcript. The missense variants affect the conserved amino acids located in the: FAD-binding domain (n = 1), insertion domain 2 (n = 1), GidA specific sequence motif (n = 5) and central helical domain (n = 8) of the MTO1 [21]. All of the variants are extremely rare and either not observed in the population databases or observed at very low frequencies. All but two variants are predicted to be damaging by *in silico* prediction tools (Table 2). Patient 13 was found to have three rare variants in *MTO1*: p.(Ala428Thr) inherited from father and p.(Val41Gly) and p.(His256Arg) both inherited from mother. While the p.(Ala428Thr) and p.(Val41Gly) are predicted damaging and have been described in other patients with MTO1 deficiency, the p.(His256Arg) variant inherited *in cis* with the p.(Val41Gly) is predicted benign by tested tools and may not contribute to the phenotype. In addition to p.(His256Arg), the splice-site variant in patient 3 has lower than expected pathogenicity score and the predicted effect on splicing is currently being tested experimentally.

**Table 2:** Overview of the genotypic data of patients with confirmed MTO1 deficiency.

Patient no	Nucleotide change	Protein change	Inheritance	In silico prediction scores	ExAC frequency
1	c.1451G > A c.1273G > A	p.Arg484Gln p.Gly425Arg	Compound heterozygous	CADD (33) SIFT (DAMAGING; 0.002) PolyPhen2(PROBABLY DAMAGING; 1.000) CADD (34) SIFT (DAMAGING; 0.001) PolyPhen2(PROBABLY DAMAGING; 1.000)	0 0.000008237
2	c.1451G > A c.1273G > A	p.Arg484Gln p.Gly425Arg	Compound heterozygous	CADD (33) SIFT (DAMAGING; 0.002) PolyPhen2(PROBABLY DAMAGING; 1.000) CADD (34) SIFT (DAMAGING; 0.001) PolyPhen2(PROBABLY DAMAGING; 1.000)	0 0.000008237
3	c.1222 A > T	p.Ile408Phe	Homozygous	CADD (29.1) SIFT (DAMAGING; 0.001) PolyPhen2(PROBABLY DAMAGING; 1.000)	0
4	c.963 A > C c.1429 C > T	p.Lys321Asn p.Arg477Cys	Compound heterozygous	CADD (24.4) SIFT (DAMAGING; 0.001) PolyPhen2(PROBABLY DAMAGING; 1.000) CADD (35) SIFT (DAMAGING; 0.000) PolyPhen2(PROBABLY DAMAGING; 1.000)	0 0
5	c.1261-5T > G c.1430G > A	p.Val422Ilefs*18 p.Arg477His	Compound heterozygous	CADD (10.5) SIFT (NA; splice-site) PolyPhen2(NA; splice-site) CADD (35) SIFT (DAMAGING; 0.001) PolyPhen2(PROBABLY DAMAGING; 1.000)	0 0.0002307
6	c.1390C > T	p.Arg464Cys	Homozygous	CADD (35) SIFT (DAMAGING; 0.000) PolyPhen2(PROBABLY DAMAGING; 1.000)	0
7	c.1232C > T	p.Thr411IIe	Homozygous	CADD (33) SIFT (DAMAGING; 0.000) PolyPhen2(PROBABLY DAMAGING; 1.000)	0.00002472
8	c.122T > G c.1282G > A	p.Val41Gly p.Ala428Thr	Compound heterozygous	CADD (26.1) SIFT (DAMAGING; 0.000) PolyPhen2(PROBABLY DAMAGING; 0.998) CADD (34) SIFT (DAMAGING; 0.002) PolyPhen2(PROBABLY DAMAGING; 1.000)	0 0.00005766
9	c.1282G > A c.402_403delTA	p.Ala428Thr p.Tyr134Terfs	Compound heterozygous	CADD (34) SIFT (DAMAGING; 0.002) PolyPhen2(PROBABLY DAMAGING; 1.000) Frameshift	0.00005766 0.000008241
10	c.631-631delG c.I282G > A	p.Gly211Aspfs*3 p.Ala428Thr	Compound heterozygous	Frameshift CADD (34) SIFT (DAMAGING; 0.002) PolyPhen2(PROBABLY DAMAGING; 1.000)	0 0.00005766
11	c.1232C > T	p.Thr411IIe	Homozygous	CADD (33) SIFT (DAMAGING; 0.000) PolyPhen2(PROBABLY DAMAGING; 1.000)	0.00002472
12	c.1232C > T	p.Thr411IIe	Homozygous	CADD (33) SIFT (DAMAGING; 0.000) PolyPhen2(PROBABLY DAMAGING; 1.000)	0.00002472
13	c.122T > G c.767A > G c.1282G > A	p.Val41Gly p.His256Arg p.Ala428Thr	Compound heterozygous	CADD (26.1) SIFT (DAMAGING; 0.000) PolyPhen2(PROBABLY DAMAGING; 0.998) CADD (10.27) SIFT (TOLERATED; 0.097) PolyPhen2(BENIGN; 0.000) CADD (34) SIFT (DAMAGING; 0.002) PolyPhen2(PROBABLY DAMAGING; 1.000)	0 0 0.00005766
14	c.1282G > A c.1858dup	p.Ala428Thr p.Arg620Lysfs*8	Compound heterozygous	CADD (34) SIFT (DAMAGING; 0.002) PolyPhen2(PROBABLY DAMAGING; 1.000) Frameshift	0.00005766 0
15	c.1282G > A c.1858dup	p.Ala428Thr p.Arg620Lysfs*8	Compound heterozygous	CADD (34) SIFT (DAMAGING; 0.002) PolyPhen2(PROBABLY DAMAGING; 1.000) Frameshift	0.00005766 0
16	c.1282G > A	p.Ala428Thr	Homozygous	CADD (34) SIFT (DAMAGING; 0.002) PolyPhen2(PROBABLY DAMAGING; 1.000)	0.00005766
17	c.1282G > A c.1430G > A	p.Ala428Thr p.Arg477His	Compound heterozygous	CADD (34) SIFT (DAMAGING; 0.002) PolyPhen2(PROBABLY DAMAGING; 1.000) CADD (35) SIFT (DAMAGING; 0.001) PolyPhen2(PROBABLY DAMAGING; 1.000)	0.00005766 0.0002307
18	c.1232C > T	p.Thr411IIe	Homozygous	CADD (33) SIFT (DAMAGING; 0.000) PolyPhen2(PROBABLY DAMAGING; 1.000)	0.00002472
19	c.1232C > T	p.Thr411IIe	Homozygous	CADD (33) SIFT (DAMAGING; 0.000) PolyPhen2(PROBABLY DAMAGING; 1.000)	0.00002472
20	c.1232C > T	p.Thr411IIe	Homozygous	CADD (33) SIFT (DAMAGING; 0.000) PolyPhen2(PROBABLY DAMAGING; 1.000)	0.00002472
21	c.1232C > T	p.Thr411IIe	Homozygous	CADD (33) SIFT (DAMAGING; 0.000) PolyPhen2(PROBABLY DAMAGING; 1.000)	0.00002472
22	c.1222A > T	p.IIe408Phe	Homozygous	CADD (29.1) SIFT (DAMAGING; 0.001) PolyPhen2(PROBABLY DAMAGING; 1.000)	0
23	c.1273G > A c.1417C > T	p.Gly425Arg p.Arg473Cys	Compound heterozygous	CADD (34) SIFT (DAMAGING; 0.001) PolyPhen2(PROBABLY DAMAGING; 1.000) CADD (35) SIFT (DAMAGING; 0.000) PolyPhen2(PROBABLY DAMAGING; 1.000)	0.000008237 0
24	c.1273G > A c.1417C > T	p.Gly425Arg p.Arg473Cys	Compound heterozygous	CADD (34) SIFT (DAMAGING; 0.001) PolyPhen2(PROBABLY DAMAGING; 1.000) CADD (35) SIFT (DAMAGING; 0.000) PolyPhen2(PROBABLY DAMAGING; 1.000)	0.000008237 0
25	c.1390C > T c.1430G > A	p.Arg464Cys p.Arg477His	Compound heterozygous	CADD (35) SIFT (DAMAGING; 0.000) PolyPhen2(PROBABLY DAMAGING; 1.000) CADD (35) SIFT (DAMAGING; 0.001) PolyPhen2(PROBABLY DAMAGING; 1.000)	0 0.0002307
26	c.1390C > T c.1430G > A	p.Arg464Cys p.Arg477His	Compound heterozygous	CADD (35) SIFT (DAMAGING; 0.000) PolyPhen2(PROBABLY DAMAGING; 1.000) CADD (35) SIFT (DAMAGING; 0.001) PolyPhen2(PROBABLY DAMAGING; 1.000)	0 0.0002307
27	c.1430G > A c.1450C > T	p.Arg477His p.Arg484Trp	Compound heterozygous	CADD (35) SIFT (DAMAGING; 0.001) PolyPhen2(PROBABLY DAMAGING; 1.000) CADD (34) SIFT (DAMAGING; 0.000) PolyPhen2(PROBABLY DAMAGING; 1.000)	0.0002307 0.0000165
28	c.1430G > A c.1450C > T	p.Arg477His p.Arg484Trp	Compound heterozygous	CADD (35) SIFT (DAMAGING; 0.001) PolyPhen2(PROBABLY DAMAGING; 1.000) CADD (34) SIFT (DAMAGING; 0.000) PolyPhen2(PROBABLY DAMAGING; 1.000)	0.0002307 0.0000165
29	c.1390C > T	p.Arg464Cys	Homozygous	CADD (35) SIFT (DAMAGING; 0.000) PolyPhen2(PROBABLY DAMAGING; 1.000)	0
30	c.1390C > T	p.Arg464Cys	Homozygous	CADD (35) SIFT (DAMAGING; 0.000) PolyPhen2(PROBABLY DAMAGING; 1.000)	0
31	c.1390C > T	p.Arg464Cys	Homozygous	CADD (35) SIFT (DAMAGING; 0.000) PolyPhen2(PROBABLY DAMAGING; 1.000)	0
32	c.1390C > T	p.Arg464Cys	Homozygous	CADD (35) SIFT (DAMAGING; 0.000) PolyPhen2(PROBABLY DAMAGING; 1.000)	0
33	c.1390C > T	p.Arg464Cys	Homozygous	CADD (35) SIFT (DAMAGING; 0.000) PolyPhen2(PROBABLY DAMAGING; 1.000)	0
34	c.1436A > T	p. Asp479Gly	Homozygous	CADD (31) SIFT (DAMAGING;0.002) PolyPhen2(PROBABLY DAMAGING; 0.998)	0.000008241
35	c.1949C > T c.722C > T	p.Thr650Ile p.Pro241Leu	Compound heterozygous	CADD (33) SIFT (DAMAGING; 0.001) PolyPhen2(PROBABLY DAMAGING; 1.000) CADD (33) SIFT (DAMAGING; 0.001) PolyPhen2(PROBABLY DAMAGING; 1.000)	0 0.000008241

Genotypes of 35 patients with MTO1 deficiency including inheritance pattern (homozygous versus compound heterozygotes), *in silico* prediction scores (CADD, SIFT and Polyphen) and frequency observed in the ExAC database (http://exac.broadinstitute.org/).

Of the 35 patients, 17 are homozygous and 13 compound heterozygous for missense variants, four are compound heterozygous for a missense and a frameshift variant and one is compound heterozygous for a missense and a predicted splice-site variant (Fig. 1 and Table 2). None of the patients have bi-allelic truncating variants (*i.e.* frameshift) suggesting that complete loss of MTO1 is unlikely to be viable in humans. To determine to what extent the *MTO1* genotype may correlate with the clinical presentation, we first grouped the patients according to the shared genotype. We observed that the four patients from three unrelated families who are compound heterozygous for the p.(Ala428Thr) variant and a frameshift variant appear to have more severe presentation: earlier than average age of presentation of clinical features at 0.04 years and shorter than average survival time at 0.24 years, further supporting the hypothesis that residual MTO1 protein function is needed for survival. Beyond the probands who are compound heterozygous for one truncating variant, we also observed that probands homozygous for the p.(Thr411Ile) variant tend to have more severe phenotype, while the probands homozygous for the p.(Arg464Cys) variant tend to have longer than average survival time.

### 3.4 Biochemical phenotypic spectrum (Table 3)

#### 3.4.1 Clinical chemistry

The most frequent biochemical feature at initial presentation was lactic acidosis, identified in 21/34 [62%; 95% CI, 45–76%] cases in whom it was measured. Eventually, once tested it was present in all patients, 35/35 (100%). The average recorded peak level of plasma lactate of 13.6 mmol/L (range 3.4 to 57.8 mmol/L). An elevated plasma alanine was documented in 21/24 [88%; 95% CI, 69–96%] cases with the average recorded level of 1346 μmol/L (range 630–6560 μmol/L). Markers of mitochondrial dysfunction, including lactate, ketones, TCA (Krebs cycle) metabolites, tyrosine metabolites, dicarboxylic acids and 3-methylglutaconate, were eventually present in the urinary organic acid profile in various patterns in every patient analyzed (14/14 [100%; 95% CI, 78–100%]) and CSF lactate was elevated in 5/6 cases [83%; 95% CI, 43–97%].

**Table 3:** Overview of the biochemical data of patients with confirmed MTO1 deficiency.

Patient no	Respiratory chain analysis	Elevated plasma lactate (mmol/L)	Elevated plasma alanine (μmol/L)	Urine analysis		CSF analysis
1	Complex I/IV def (muscle)	Y (4.4)	Y (657) (ref: 148–475)			
2	Complex I/IV def (muscle)	Y (4.3)	Y (711) (ref: 148–475)			
3	Normal (fibroblasts)	Y (8)	Y			Elevated lactate (3.3 mmol/L)
4	Complex I/IV def (muscle)	Y (18)	N	Elevated lactate, pyruvate, ketones		
5	Complex I/IV def (muscle)	Y (11.1)	Y (800)			
6	Complex I//III/IV def (muscle)	Y(3.7)	N	Elevated lactate, pyruvic acid, 2/3-hydroxybutyrate, 2-hydroxybutyrate, homovanillic acid, 4-hydroxyphenyllactate, acetoacetate, N- acetyltyrosine, phenyl pyruvate		
7	Complex I/IV def (muscle)	Y (29)	Y	Elevated lactate, ketones		
8	Complex I/IV (muscle)	Y (11.2)	Y (792)	Elevated, lactate, 3-methylglutaconate, ketones, 4-hydroxyphenyllactate, phenylpyruvate, increased TCA intermediates		
9	Complex I/IV (muscle)	Y (6.1)	Y	Elevated, lactate, pyruvate, 4-hydroxyphenyllactate, phenylpyruvate, ketones, DCA, 2-hydroxybutyrate, dihydroxyhexanoate, ketones, phenylpyruvate, TCA intermediates		
10	Complex I/IV def (muscle)	Y	Y (1123)			
11	Complex I/IV def (muscle)	Y (57.8)	NR	Elevated lactate		
12	Complex I/IV def (muscle)	Y (23.5)	N	Elevated lactate		
13	Complex I/IV def (muscle)	Y				
14	Complex III/IV def (fibroblasts)	Y (13)				
15	Complex I/IV def (muscle)	Y (17.9)				
16	Complex I/IV def (muscle)	Y (5.5)				
17	Complex I/IV def (muscle)	Y	Y	Elevated urinary lactate, pyruvate, TCA cycle intermediates		
18	Complex I/IV def (muscle)	Y (14.6)	Y	Elevated urinary lactate, 3-methylglutaconate, TCA cycle intermediates		
19	N/D	Y				
20	Complex IV def (muscle)	Y				
21	Complex IV def (muscle)	Y	Y			
22	Complex IV def (fibroblasts) Complex I def (muscle)	Y	Y			
23	Complex I//III/IV def (muscle)	Y (15)	Y (630)			Elevated lactate (3.2 mmol/L)
24	Complex I//III/IV def (muscle)	Y (15)	NR			Elevated lactate (2.6 mmol/L)
25	Complex I/IV def (muscle)	Y (3.4)	Y(771)	Elevated TCA cycle intermediates, ketones		Elevated lactate (4.93 mmol/L);
26	N/D	Y (11.2)	Y	Elevated TCA cycle intermediates, ketones		
27	Normal (fibroblasts)	Y (28)	Y (6560)	Elevated urinary lactate, pyruvate, 2/3-hydroxybutyrate, acetoacetate		
28	Complex I/IV def (muscle)	Y (15.5)	Y (N/A)	Elevated lactate, pyruvate, 2/3-hydroxybutyrate, 2-hydroxyisovalerate, 2-oxoisovalerate, 2-oxo-3-methylvalerate, acetoacetate, 4-hydroxyphenyllactate, 4-hydroxyphenylpyruvate, hydroxydecanoic acid		
29	Complex I/IV def (muscle)	Y (3.7)	Y (568)			Decreased CSF 5-methyltetrahydrofolate (29 nmol/l), increased homovanillic acid (579 nmol/L)
30	Complex I/III/IV def (muscle)	Y (6.4)	Y (998)			
31	Complex I/IV def (muscle)	Y				
32	Complex I/III/IV def (muscle)	Y (6.5)				
33	Complex I/III/IV def (muscle)	Y				
34	Complex I/IV def (muscle)	Y (19)	Y (1196)	Elevated lactate, 2-hydroxybutyrate, 4-hydroxyphenyllactate		Normal lactate
35	Normal (muscle/fibroblasts)	Y (3.4)	Y			Elevated lactate (2.5 mmol/L)

Biochemical phenotype of 35 patients with MTO1 deficiency including respiratory chain analysis, plasma lactate levels, plasma alanine, urine analysis and CSF analysis.

#### 3.4.2 Respiratory chain analysis

Respiratory chain enzyme (RCE) analysis in muscle was performed in 30 patients. Complex IV was deficient in 28/30 cases and 27/30 cases showed evidence of combined deficiency. Of the combined deficiencies, complex I and IV deficiency were most commonly seen (20/30 cases) while combined complex I, III and IV deficiency, was present in 6/30 cases. In fibroblasts, RCE analyses varied from normal in 4/10 [33%] to a single complex deficiency in 2/10 [20%], while a combined complex deficiency was present in 4/10 [40%]. Also in this tissue, the most commonly observed biochemical defect was a combined complex I and IV deficiency.

### 3.5 Treatments

Various combinations of vitamin/anti-oxidant supplements (the ‘mitochondrial cocktail’) according to each institution clinical practice were administered. Of the 35 patients, 8 did not have any treatment directed towards primary mitochondrial dysfunction and for 2 information is unavailable. Supportive treatments, not directed at primary mitochondrial dysfunction such as inotropes and tube feeding, were not recorded. For the remaining patients, 14/25 [56%] cases were commenced on L-carnitine, 17/25 [68%] on co-enzyme Q10, 6/25 [24%] on vitamin C, 4/25 [16%] on vitamin E, 11/25 [44%] on riboflavin and 5/25 [20%] on thiamine which had no appreciable effect. Also, dichloroacetate was administered to 8/25 [32%] patients with the intent of lowering lactate levels. The ketogenic diet was trialled in 5/22 [23%] patients, 3 of whom were deemed unresponsive. Only one patient (patient 1) showed a clear clinical improvement while in one other patient (patient 4) seizures were initially better controlled after the start of the diet, but at 8 years, different seizures types developed (drop attacks) and the diet fat ratio was reduced. Visual deterioration occurred soon after onset of ketogenic diet in patient no. 4 and in retrospect, this symptom was unlikely related to the diet, but rather the result of a secondary complication of MTO1 deficiency.

## 4 Discussion

The current series of 35 cases with bi-allelic mutations in *MTO1*, which encodes a ubiquitously expressed enzyme necessary for optimization of mtDNA-dependent protein synthesis, is to our knowledge the largest to date. Review of their clinical features confirmed as hallmarks of this primary mitochondrial disease developmental/cognitive impairment, lactic acidosis and complex IV deficiency (± other deficiencies) in muscle (http://www.proteinatlas.org/ENSG00000135297-MTO1/tissue). Two patients are presented in detail to illustrate the wide phenotypic spectrum of MTO1 deficiency, i.e. that a typical clinical presentation for this condition does not exist and thus must be considered in patients with a variety of features. Although early reports suggested that HCM at a young age to be pathognomonic for MTO1 deficiency, HCM was absent in both presented adolescent siblings. A further four cases of MTO1 deficiency without cardiomyopathy at ages 0.66 to 22 years, one published and three unpublished, were identified in our review. Together, these six cases prove that HCM is not a hallmark clinical feature of this condition; in fact it was only reported as a presenting feature in approximately half of cases (see Table 1). Although in MTO1 deficiency the cardiomyopathy is usually hypertrophic, one patient developed a dilated form (patient 12) which we recognize may be a progression from HCM. As is common in rare diseases, the phenotype of MTO1 deficiency appears more variable than initially thought.

Other commonly occurring clinical features identified were feeding difficulties, FTT, hypotonia and ocular pathology. Frequently occurring biochemical characteristics include elevated plasma alanine and mitochondrial markers in urine. This review also identified a broad phenotypic spectrum for MTO1 deficiency from a severe, rapidly progressive, ultimately fatal presentation in the neonatal period to that of an 8-year-old presenting with ID, fatigability and clumsiness and surviving to adulthood to that of a 19-year-old male who presented at age 1 month with feeding difficulties, poor ocular fixation and HCM with reversal of many of his symptoms with by 1 year. At 19 years this patient has a recurrence of HCM but has normal scholastic performance (Table 1).

The cohort of patients reviewed here further delineates the genotype-phenotype relationship in MTO1 deficiency [7]. Consistent with previous reports of an early-onset for the majority of mitochondrial diseases involving the respiratory chain [22], almost 50% of our cohort presented in the first two days of life and the remainder presented by end of childhood, which is unsurprising for a deficiency of an enzyme so ubiquitously expressed and involved in such a fundamental process as posttranscriptional modification of specific mt-tRNAs [4], [5]. Of those who presented in the first two days of life, 50% were deceased by two years suggesting a positive correlation between early neonatal presentation and an unfavourable prognosis, an observation supported by Martin et al. [23]. Importantly, all of the patients who are compound heterozygous for a missense variant and a frameshift variant had early presentation and shorter than average survival time; none of the 35 cases had bi-allelic truncating *MTO1* alleles, suggesting that residual MTO1 protein function is needed for survival in humans. An unexpected observation was that the sex ratio of those who are deceased is skewed toward males, the cause of which is unexplained. To date cases have been reported in 11 different countries suggesting that MTO1 deficiency is probably a pan-ethnic condition (see Table 1).

The most frequent neurologic features of primary mitochondrial disease are muscle weakness with hypotonia, followed by clinical or imaging features of central neurological disease and cognitive impairment/decline [22], [24], [25], [26]. In the presented cohort, clinical presentation for the majority was during the neonatal period when global developmental delay is difficult to assess. Virtually all of the patients for whom clinical information was available at a later age did show an intellectual developmental disorder and/or other neurologic impairment. Seizures were documented in approximately one-third of cases while abnormalities of brain structure (including anomalies of the claustrum and surrounding capsule, thalami, subcortical white matter, cerebellar peduncles and the corpus callosum) and an elevated lactate peak on spectroscopy were reported in more than three-quarters of cases who underwent brain imaging. (Table 1 and Supplementary Table 1).

Interestingly, the phenotype differs between MTO1 and other proteins involved in post transcriptional modification of specific mt-tRNAs, such for example tRNA 5-methylaminomethyl-2-thiouridylate methyltransferase deficiency (TRMU) [27], [28]. While TRMU mutations may result in severe infantile hepatopathy or renal failure neither hepatopathy nor renal pathology are common features in MTO1 deficiency with each observed in fewer than 10% of cases [29], [30], [31]. These may however be features that will develop later in life.

Although the diagnosis of MTO1 deficiency may be suggested by the presence of mitochondrial markers, such as plasma lactate, amino acids, urinary mitochondrial markers and CSF lactate; these are not specific (Table 3). Accordingly, the majority of cases were diagnosed by whole or targeted exome sequencing rather than single gene tests. Respiratory Chain Enzyme (RCE) analysis of muscle is a sensitive (though not specific) test for MTO1 deficiency with enzyme deficiencies in all cases known to date.

Given the role of MTO1 as an optimizer of mtDNA translation it is not surprising that *MTO1* mutations can be associated with impaired mitochondrial protein synthesis and result in any combination of RC deficiencies, from isolated complex IV deficiency to a combined complex I and complex IV deficiency, the most commonly observed biochemical signature. Complex I, III and IV deficiency, or isolated deficiencies of complex I or complex IV were seen in a few patients. This may be explained by the destination of the 13 mtDNA encoded proteins; seven are core subunits of complex I, three of complex IV, two of complex V (although this OXPHOS component cannot be easily measured in frozen diagnostic muscle samples) and one of complex III; complex II is entirely nuclear-encoded. Importantly the high number of normal results in fibroblasts indicates that normal RCE analysis in this cell type *do not* exclude the possibility of a MTO1 defect. Given the lack of specificity and invasiveness of some of these tests (muscle biopsy) targeted WES approach may be the single most effective diagnostic approach to MTO1 deficiency which is illustrated by the two presented cases.

Since the first diagnosed case of MTO1 deficiency in 2012, many cases have been trialled on several therapies such as L-carnitine, coenzyme Q10, vitamin C, riboflavin, thiamine, dichloroacetate (DCA) and/or vitamin E. Despite the use of multiple variations of this “mitochondrial cocktail”, documented objective improvement was rare. As MTO1 is a FAD moiety-containing enzyme, riboflavin supplementation was investigated as a possible treatment without observed success in either respiratory chain or fibroblast oxygen consumption analyses [6]. One such case where clinical and biochemical improvement was noted was in patient no. 16 (Table 1). In this case DCA is thought to have resulted in a marked improvement in LA without side effects reported. HCM also improved resulting in a normal ECHO at 9 months and a normal scholastic performance at 19 years [6]. The authors recognise that DCA is expected to improve LA without a clear effect on seizures or neurological outcome [32]. Whether or not DCA had a direct positive effect on HCM in patient no. 16 is uncertain and the improvement may have been part of the natural disease course in this case. One of the three patients with *MTO1* deficiency described by Martin et al. is also reported to have improved on therapy. This report however should be viewed cautiously as this patient also had vitamin B12 deficiency and secondary hyperhomocystinemia; the improvement might have been due to treatment of this deficiency rather than the MTO1 mitochondriopathy as the lactic acidemia persisted [33]. Clinical improvement was recorded in one of five patients trialled on ketogenic diets. At age 19 months persistently high lactate levels (between 3 and 7 mmol/l) were present. After several months on a moderate ketogenic diet the lactate levels normalized and they remain normal at the time of publication. Improvement in motor development was observed, as the patient learned to walk soon after starting the diet and at 4 years of age he was able to walk several hundred meters despite remaining ataxic. Speech development remained poor with the expressive domain more severely affected than receptive. His initial mild cardiomyopathy resolved completely which the authors recognize may have been the cause of his lactic acidemia and he never developed seizures. Our female index initially also showed an improvement on a ketogenic diet but subsequently her seizures worsened. Thus the current data are insufficient to draw conclusions about the effectiveness of the ketogenic diet; given the clinical improvement in the two patients described above, it may be worth cautiously considering this treatment in MTO1 deficient patients with seizures, especially in light of the report of amelioration of the defects in oxidative phosphorylation observed in MTO1 KO mice on a ketogenic diet [11]. Of note Bartsakoulia et al suggests that N-acetyl-cysteine supplementation may have a beneficial effect on mitochondrial translation in MTO1 deficient fibroblasts and this is a future potential treatment [34].

The authors acknowledge several limitations in the study. Although features such as LA and HCM seem to be the clinical hallmark of MTO1 deficiency, caution must be observed given the possible ascertainment bias introduced by the preselection based on the presence of these symptoms. Also, because the ages of the patients differ with varied follow-up duration and quality, symptoms occurring later in life may go unreported. An unexpected observation was that the sex ratio of those who are deceased is skewed toward males. This is unusual for a recessive condition and the authors recognize that there is no obvious biological reason for this and it may be an artefact of a small cohort. Although great care was used in compiling the data kindly provided by clinicians and scientists in different parts of the world, the authors recognize a potential limitation in the study was the lack of usage of uniform terminology for clinical and biochemical signs and symptoms. We strongly advocate for use of Human Phenotype Ontology (HPO) (http://human-phenotype-ontology.github.io/) terms to enhance standardization, which will facilitate and improve such studies in the future. Furthermore, in the literature and lab reports, multiple transcript variants encoding at least 3 different MTO1 isoforms are used which has a potential for confounding reporting and interpretation. Isoform a (NM_012123.3) was chosen and in those papers where isoform b (NM_133645, NP_598400) or c (NM_001123226, NP_001116698) was used, the amino acid position was corrected according to isoform a, for consistency in this study.

In summary, the mitochondrial disease knowledgebase is expanded with this report on the largest cohort of MTO1 deficient patients to date and discussion about the phenotype (clinical and biochemical), genotype, natural history, outcomes and treatments. The features of MTO1 deficiency are non-specific which can make accurate diagnosis difficult, often requiring exome sequencing or gene panel analysis. We do recommend that physicians should consider this disease in patients with LA, developmental/cognitive delay and other features of mitochondrial disease. The report of three cases (the two presented siblings and case no. 35) without cardiomyopathy at the adolescent age/early twenties demonstrates that it is not an obligatory feature of the disease, which is important for differential diagnostics. RCE analysis on muscle rather than fibroblasts is recommended as the latter can yield false negative results. Once further MTO1 deficiency patients are diagnosed/identified, development of phenotypic subgroups may become a possibility. In general, early diagnosis is important for genetic counselling, prognostication, screening for organ involvement, ending the diagnostic odyssey and considering disease modifying interventions, such as the ketogenic diet and, in the future, new therapies such as exosome-based delivery [35].

The following are the supplementary data related to this article.

Supplementary Table 1 Neuroimaging results. Brain MRI and MR spectroscopy results on the 35 patients with confirmed MTO1 deficiency Supplementary Table 1
