# Supplementary material for: The genotypic and phenotypic spectrum of MTO1 deficiency
Source: Mol Genet Metab. 2018 Jan;123(1):28–42. doi: 10.1016/j.ymgme.2017.11.003 (PMC5780301; doi:10.1016/j.ymgme.2017.11.003)
Supplement: Supplementary Table 1 — Neuroimaging results. Brain MRI and MR spectroscopy results on the 35 patients with confirmed MTO1 deficiency [file mmc1.docx]

| Patient No | Neuroimaging MRI/MRS /US |
| --- | --- |
|  |  |
|  |  |
| 1 | MRI at 8 yr - abnormal signal intensity in midline of the midbrain and chiasm.  MRI at 11 yr - multiple areas of hyperintense T2/FLAIR signal within the cerebral peduncles, basal ganglia and cortex (resolved on repeat study). |
| 2 | MRI – Normal. |
| 3 | MRI- Bilateral basal ganglia lesions. [H+]-MR Spectroscopy - increased lactate peak. |
| 4 | MRI- Basal ganglia hyperintensity. |
| 5 | MRI- Corpus callosum hypoplasia. |
| 6 | MRI - Generalised cortical involvement and minimal cerebellar atrophy. |
| 7 | MRI/MRS –Normal. |
| 8 | MRI- Abnormal signal in the thalami, mid-brain, pons. |
| 9 | MRI- Abnormal signal in subthalamic nuclei, substantia nigra, tegmentum of midbrain, superior and inferior cerebellar peduncles at the level of medulla and medullary pyramids. |
| 10 |  |
| 11 | MRI- Normal. |
| 12 |  |
| 13 |  |
| 14 |  |
| 15 |  |
| 16 | MRI – Normal. |
| 17 | MRIs - Bilateral hyperintensities in the capsulae surrounding the claustra. |
| 18 | MRI- Symmetrical, bilateral abnormal signals in fornices, globus pallidus, thalamus, subthalamic nucleus, substantia nigra, dorsal mesencephalon, pons and to a lesser extent dentate nuclei of the cerebellum. [H+]-MR Spectroscopy - increased lactate peak. |
| 19 |  |
| 20 |  |
| 21 |  |
| 22 |  |
| 23 | MRI - Severe frontotemporal atrophy, bilateral increased signal intensity in thalamus upper part. |
| 24 | MRI - Bilateral hyperintensity in thalami. |
| 25 | MRI - Symmetrical bilateral abnormal signals in thalamus, substantia nigra, mesencephalon and pons. [H+]-MR Spectroscopy lactate peak detected. |
| 26 |  |
| 27 |  |
| 28 |  |
| 29 | MRI – Normal. |
| 30 | MRI - Arachnoid cyst on the pontocerebellar right side. |
| 31 | MRI - Bilateral signal hyperintensity involving dentate nuclei and a precentral cortex. |
| 32 |  |
| 33 |  |
| 34 | MRI/ MRS - Normal |
| 35 | MRI - Mild Increase in periventricular white matter signal. [H+]-MR Spectroscopy – Normal. |
